# INM004: Polyclonal Neutralizing Antibodies Against Shiga Toxin as a Treatment for Hemolytic Uremic Syndrome

**DOI:** 10.3390/toxins17060282

**Published:** 2025-06-05

**Authors:** Marta Rivas, Mariana Pichel, Vanesa Zylberman, Mariana Colonna, Marina Valerio, Carolina Massa, Romina Pardo, Andrés E. Ciocchini, Santiago Sanguineti, Ian Roubicek, Linus Spatz, Fernando Alberto Goldbaum

**Affiliations:** 1 Inmunova S.A., Av. 25 de Mayo 1021, San Martín, Buenos Aires 1650, Argentina; mrivas@inmunova.com (M.R.); mpichel@inmunova.com (M.P.); vzylberman@inmunova.com (V.Z.); mcolonna@inmunova.com (M.C.); mvalerio@inmunova.com (M.V.); cmassa@inmunova.com (C.M.); rpardo@inmunova.com (R.P.); ssanguineti@inmunova.com (S.S.); linus@inmunova.com (L.S.); 2 Consejo Nacional de Investigaciones Cientificas y Tecnicas (CONICET), Godoy Cruz 2290, Ciudad Autonoma de Buenos Aires 1425, Argentina; 3 Instituto de Investigaciones Biotecnológicas, Universidad Nacional de San Martín (UNSAM), Escuela de Bio y Nanotecnologías, UNSAM Campus Miguelete, 25 de Mayo y Francia, San Martín 1650, Argentina

**Keywords:** Shiga toxin, hemolytic uremic syndrome, STEC-HUS, diagnosis, immunotherapy treatment, vaccine development

## Abstract

Hemolytic uremic syndrome (HUS) is a thrombotic microangiopathy characterized by microangiopathic hemolytic anemia, thrombocytopenia, and acute kidney injury (AKI). Shiga toxin (Stx)-producing *Escherichia coli*-associated HUS (STEC-HUS) is one of the leading causes of AKI in children. Approximately 1.5 to 3% of children die during the acute phase, and about 30% experience long-term renal sequelae. Argentina has the highest incidence of STEC-HUS globally. Given the prominent role of Stx in its pathophysiology, STEC-HUS is considered more of a toxemia than a bacterial disease. Stx transport occurs before and after the STEC-HUS onset, allowing for the distinction between an early toxemia phase and an advanced toxemia phase. In this review, we present our efforts to develop INM004, an anti-Stx treatment aimed at ameliorating or preventing the clinical consequences of STEC-HUS. We describe the protein engineering that facilitated this development and the clinical path to demonstrate the safety and efficacy of INM004. This immunotherapy could represent a new step in the treatment of STEC-HUS, which could potentially prevent long-term damage. If phase 3 trials are successful, earlier and broader use of INM004 is envisioned. We also discuss the potential impact of INM004 therapy, targeted vaccination strategies, and new diagnostic tools for this disease.

## 1. STEC-HUS, a Neglected Disease

Hemolytic uremic syndrome (HUS) is a form of thrombotic microangiopathy (TMA) characterized by microangiopathic hemolytic anemia, thrombocytopenia, and acute kidney injury (AKI) [1]. According to Loirat et al. [2], HUS can be classified as: (i) HUS of infectious origin, associated with Shiga toxin (Stx)-producing *Escherichia coli* (STEC) infection or STEC-HUS, formerly called typical, postenteric or D+ HUS. Other infectious forms have been associated with *Shigella dysenteriae* Type 1, *Streptococcus pneumoniae*, Influenza A H1N1 or HIV; (ii) HUS related to complement abnormalities, also known as atypical HUS (aHUS), not associated with diarrhea; (iii) HUS associated with coexisting diseases (bone marrow transplantation, solid organ transplantation, cancer, autoimmune disorders); (iv) HUS of unknown etiology. STEC-HUS is the most common form, representing 90% to 95% of cases, while aHUS constitutes 5% to 10% of cases [3]. STEC-HUS is one of the leading causes of AKI in children worldwide. Approximately 1.5 to 3% of children die during the acute phase of the disease, and about 30% experience long-term renal sequelae [4]. STEC-HUS is a life-threatening orphan disease, with most cases occurring during warmer months [5]. Currently, there is no specific treatment capable of preventing or controlling kidney, hematological or other organ damage caused by Stx, and management remains supportive. In this review, we focus on the key role of Stx in triggering this disease and the efforts we have made to develop an anti-Stx treatment to ameliorate or prevent the clinical consequences of STEC-HUS.

We outline the journey from protein engineering to increase the immunogenicity of the toxin, detailing the strategic decisions made to shape our experimental drug and the challenging path of clinical development for INM004. Finally, we highlight the details of the last step of this journey: the ongoing phase 3 clinical trial aimed at assessing the efficacy of this treatment. This work emphasizes the significance of developing targeted therapies like INM004 for treating an orphan disease such as STEC-HUS.

## 2. Epidemiology of STEC-HUS

In a systematic review conducted to estimate the annual number of STEC-associated diseases globally, data from 21 countries were analyzed, representing a cumulative population of 2.1 billion (approximately 30% of the world’s population) from 1990 to 2012 [6]. The authors estimated that STEC causes 3890 cases of HUS annually, with 1630 (42%) cases occurring in patients aged 0 to 4 years, 720 (18%) in those aged 5 to 15 years, 720 (18%) in patients aged 16 to 59 years, and 530 (14%) in those older than 60 years.

Over the last few decades, surveillance networks have been created at regional and international levels, with the aim of improving knowledge of STEC and the epidemiology of other foodborne pathogens, as well as promoting rapid recognition of outbreaks. This is the case with Foodnet in North America, the European Center for Disease Prevention and Control (ECDC), and PulseNet International [7,8]. Nevertheless, there are still differences among the regions regarding the mandatory notification of STEC infections, as outlined below.

In the U.S., STEC is estimated to cause approximately 265,000 cases annually, ranging from uncomplicated diarrhea to HUS [9]. According to the FoodNet Network report, the incidence of HUS in subjects under 18 years of age was 0.6 cases/100,000 subjects in the period 2020–2021 [10].

In Europe, the notification of STEC infections is mandatory in all countries except in Belgium, France, and Luxembourg, where notification is voluntary, and in Italy, where it is based on another type of system. A total of 29 EU countries reported 8565 confirmed cases of STEC infections in 2022. The overall reporting rate was 2.5 cases/100,000 population; Denmark, Germany, and Ireland had the highest rates. A total of 568 cases of HUS were reported, mainly affecting children under 14 years of age: 60% in the 0–4 age group and 24% in the 5–14 age group [11].

New Zealand reports an annual infection rate of 3.3/100,000 persons, whereas Australia reports only 0.4 cases/100,000 persons [12].

In Latin America, surveillance systems for STEC infections and HUS differ in each country. The estimated annual incidence of HUS in Chile and Uruguay is fewer than 3 cases/100,000 children under 5 years of age [13]. In Peru, the incidence of HUS was 0.42 and 0.67 cases/100,000 children under 5 years of age in 2015 and 2022, respectively [14]. Argentina has the highest incidence of STEC-HUS worldwide. In this country, the disease has been endemic and notifiable since 2000, representing approximately 95% of all HUS cases [15]. It is one of the most common causes of AKI, with nearly half of the patients requiring dialysis, and a frequent cause of chronic kidney disease (CKD), being responsible for 9% of kidney transplants in children and adolescents [16]. In the period 2012 to 2022, the median number of new HUS cases reported per year to the National Health Surveillance System was 358, ranging from 402 cases in 2012 to 304 cases in 2022. The annual incidence was 6.03 cases per 100,000 children under 5 years of age in 2022 [17]. In line with what has been reported worldwide [6], epidemiological surveillance data in Argentina show that the most affected groups are children under 5 years of age, with the highest frequency observed in the 24–48-month age group, followed by the 12-month age group [17].

In Table 1, the incidence of HUS in different countries is shown.

STEC isolates are characterized by serogroup (O antigen) and genotype (genes associated with the virulence factors *stx*, intestinal adhesion *eae,* and enterohemolysin *ehxA*). The frequency of STEC serogroups varies depending on the region. While serogroup O157 has been identified as a prevalent cause of STEC-HUS worldwide, the use of new diagnostic methods has led to the identification of other non-O157 serogroups associated with the disease, including O26, O103, O111, O121, and O145 [5,23,24,25,26,27]. In the U.S. and Argentina, O157 continues to be the predominant serogroup, whereas in Europe, O26 has been reported as the most frequent serogroup in HUS cases in France, Italy, and Denmark [28]. Moreover, an unusual O80 serogroup is currently emerging in Europe, mainly in France [29]. Although the vehicles of transmission are similar for different serogroups, meat consumption has been the most commonly reported source in STEC O157 outbreaks [5].

In 2011, a Stx-EAEC O104:H4 strain caused an outbreak with more than 4000 infections, including 900 HUS cases and 50 deaths in Germany [30]. Since that large outbreak, attention has been focused on the surveillance of this and other new *E. coli* pathotypes [31].

## 3. Shiga Toxin and Pathogenesis

The production of Stx is the main virulence factor, and two families, named Stx1 and Stx2, have been described [32]. Stx has two major subunits: one A subunit that binds non-covalently to a pentamer of five identical B subunits. Subunit A damages the eukaryotic ribosome and stops protein synthesis in target cells. Pentamer B binds to the cell receptor, globotriaosylceramide (Gb3), which is found primarily on endothelial cell membranes. Renal microvascular endothelial cells are the primary target of Stx, but cells in the renal glomerulus (podocytes and mesangial cells) and extraglomerular epithelial cells of the kidney (proximal tubule and collecting duct cells) are also sensitive to Stx [33]. Several subtypes of Stx1 (Stx1a, Stx1c, Stx1d) and Stx2 (Stx2a, Stx2b, Stx2c, Stx2d, Stx2e, Stx2f, Stx2g) have been described [32,34], differing in their virulence [25,34]. Furthermore, the risk of developing HUS is higher in patients infected with Stx2-producing strains [35,36,37]. Stx1-producing strains alone are considered low risk, usually associated with uncomplicated diarrhea and rarely with HUS [5].

STEC-HUS is an acute disease that is preceded by a prodromal period of diarrhea. When STEC-contaminated food or water is consumed, STEC colonizes the colonic epithelium with the formation of a typical attachment/effacement lesion (A/E lesion) of the microvilli of the brush border of epithelial cells, in an intimate binding of the pathogen to the host enterocyte [38]. This leads to a breakdown of the intestinal barrier, disruption of the bidirectional flow of water and electrolytes, the appearance of non-bloody diarrhea lasting 1 to 3 days, and intestinal inflammation. Subsequently, in approximately 90% of cases, bloody diarrhea occurs due to the secretion of Stx and its transport from the luminal space into the systemic circulation, with damage at the intestinal microvascular level. Stx is released into circulation and reaches the vascular endothelial cells of the target organs, binding to the specific receptor Gb3, resulting in microvascular endothelial damage. In addition, lipopolysaccharide (LPS), released into the gut by STEC, can enter the systemic circulation and promote an inflammatory response that contributes to the pathogenesis of kidney injury [39].

In patients who develop HUS, renal and hematologic manifestations occur between 5 and 13 days after the onset of diarrhea, with a median of one week [40]. Thus, during STEC infection, localized intestinal disease is observed in the first stage, and subsequently, a systemic disease is caused by the dissemination of Stx to organs far from the site of release. These two stages do not always manifest clinically in the same patient, as only 10% to 15% of children with STEC infection progress to HUS [40,41,42,43].

Stx transport occurs before and after the onset of STEC-HUS, so it is possible to distinguish between an early toxemia phase (associated with localized intestinal disease) and an advanced toxemia phase (associated with the onset of HUS). Once in the bloodstream, Stx migrates to target organs, primarily the kidney and often the brain, where it binds to endothelial cells via the Gb3 receptor. Subsequently, the toxin is internalized and translocated to the *trans*-Golgi network, and then to the endoplasmic reticulum, where the catalytically active A1 fragment of the A subunit (A1 + A2) is released into the cytosol. There, it exerts its cytotoxic action by inhibiting protein synthesis and inducing apoptosis [44]. Inflammatory phenomena, with the production of cytokines and chemokines (interleukin-6, interleukin-8, tumor necrosis factor α, and interleukin-1ß), and thrombogenic phenomena are also triggered, obstructing the fine capillaries of the renal glomeruli due to the edema of the endothelial cells and the accumulation of platelets, fibrin, and fragments of red blood cells, thereby compromising the glomerular filtration rate (GFR). In addition, there is growing evidence that Stx not only acts at the level of the endothelial cells of the glomeruli but also causes lesions in other renal structures due to the presence of Gb3 in the epithelial cells of the renal tubules [5,45,46].

Stx circulates primarily bound to polymorphonuclear cells (PMNs), especially neutrophils, or in microvesicles derived from these cells, to the kidney or other target organs. To a lesser degree, Stx is transported associated with membranes or inside microvesicles derived from other blood cells, such as platelets, other types of white blood cells (such as monocytes), and red blood cells [47,48,49,50]. Stx binds to human PMN cells by interacting its A subunit with the innate immune Toll-like receptor 4 (TLR4) [51], leaving the B subunit exposed in the extracellular space. TLR4 binds to Stx with a 100-fold lower affinity than the toxin for its Gb3 receptor, explaining the role of neutrophils in the transport and transfer of Stx from the intestinal lumen to cells in the glomerular vasculature [52,53]. In patients with HUS, TLR4 expression on the surface of neutrophils increases within 24 h of diagnosis [51].

Endothelial damage is the key in the sequence of events that lead to the microangiopathic process of HUS. The Stx/Gb3 complex triggers signaling events that result in vascular dysfunction, leukocyte recruitment, platelet thrombus formation, and fibrin deposition [54,55]. In addition, changes in shear stress caused by the renal microvascular structural damage associated with lumen narrowing promote the persistence of endothelial damage, platelet activation, and microvascular thrombosis [55]. Glomerular thrombi and tubular epithelial cell damage decrease GFR and cause renal damage [56], which can manifest with varying degrees of severity, including the presence of hematuria, increased serum creatinine (sCr), decreased GFR, and oligoanuria.

Thrombocytopenia is an important clinical manifestation of STEC-HUS. Platelets are consumed in small thrombi that can occlude the microcirculation of various organs, including the kidney. During the acute phase of the disease, circulating platelets degranulate, with an altered aggregation response [55].

Hemolysis results from the mechanical fragmentation of red blood cells that pass through the microvasculature partially occluded by platelets and fibrin. Red blood cells appear as helmet cells or schistocytes, which have diagnostic value if they are found in the peripheral blood smear. Markers of hemolysis include elevated lactate dehydrogenase (LDH) levels [57].

## 4. From Bench to Bedside: How Protein Engineering Helped to Create a New Treatment

### 4.1. BLS as a Perfect Scaffold to Stabilize and Increase the Immunogenicity of StxB Subunits

The use of polymeric scaffolds to present multiple copies of molecules is being explored to enhance the immunogenicity of antigens in vaccine development [58,59,60]. Virus-like particles (VLPs), which are highly immunogenic proteins composed of a repetitive array of subunits that are marginally immunogenic in the monomeric form, are a well-known example of this approach [61]. Smaller oligomeric scaffolds than VLPs could be beneficial for the polyvalent presentation of proteins. *Brucella* spp. lumazine synthase (BLS) is an enzyme that catalyzes an intermediate step in the biosynthesis of riboflavin in bacteria, fungi, and plants [62,63]. Its decameric structure is made up of 18 kDa subunits organized into dimers of pentamers [62,64,65]. It is a powerful immunogen capable of eliciting strong humoral and cellular responses in animals inoculated either with the protein or with a plasmid coding for its sequence [66,67]. The immunogenic properties of BLS are attributed to its oligomeric configuration, its ability to activate dendritic cells [68], and its notable thermodynamic stability and resistance to proteolytic degradation [62], which may extend its half-life within host organisms.

### 4.2. Engineering of BLS-Stx

**Figure 1:**
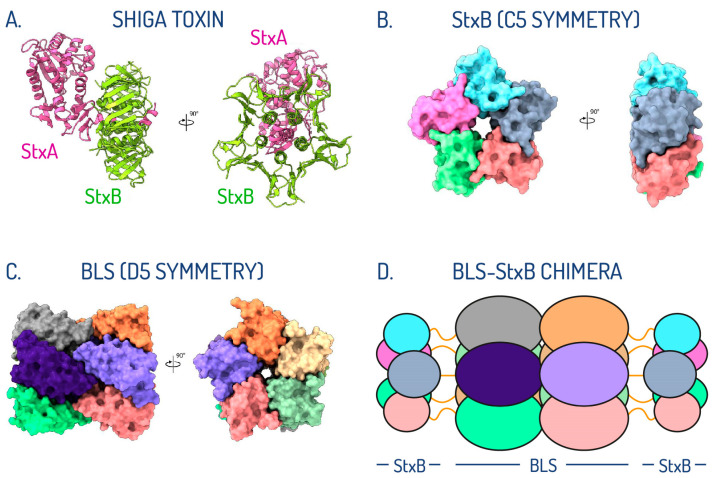
Oligomeric symmetry shared between StxB subunit and BLS. (**A**) Crystallographic structure in ribbon representation of Stx2 with the StxA and StxB subunits colored in pink and green, respectively. (**B**) Crystallographic structure in surface representation of the pentameric arrangement of the Stx2B subunit, colored by chains. (**C**) Crystallographic structure in surface representation of BLS assembled as a dimer of pentamers, colored by chains. (**D**) Schematic representation of the postulated di-pentameric model of BLS engineered with Stx1B or Stx2B at the amino terminus. Linkers connecting both proteins are depicted as orange lines (adapted from Cristófalo et al., 2025 [83]).

Despite the differences in toxicity, the subtypes of Stx share a similar structural arrangement, in which a single A subunit (StxA) is assembled with five B subunits (StxB) (Figure 1A) [69,70,71,72,73,74]. StxA is an enzymatically active 32 kDa monomer composed of two peptides—A1 (27.5 kDa) and A2 (4.5 kDa)—that inhibits protein synthesis in the affected cell. StxB is a homopentamer with a total molecular mass of 38.5 kDa (Figure 1B), responsible for the internalization of the toxin by binding to the cell surface Gb3 receptor.

The role of Stx makes STEC-HUS a toxemia rather than a systemic bacterial disease. Therefore, the neutralization of Stx in compromised patients by passive immunization with antibodies offers a promising approach for treating the disease. In this context, the B subunit of Stx2 (Stx2B) emerges as a particularly attractive target for developing neutralizing antibodies, given its interaction with Gb3 and the high prevalence of Stx2 in patients with HUS. However, the potential of Stx2B as a target is limited by its poor immunogenicity [75], likely due to its unstable pentameric structure [76].

With the aim of stabilizing the oligomeric assembly of Stx2B, we devised a rational protein engineering strategy focused on the use of the lumazine synthase from *Brucella* sp. (BLS) as a structural scaffold [62,77]. By exploiting the shared oligomeric symmetry between the two proteins, we engineered Stx2B at the amino termini of BLS, creating a 254 kDa BLS-Stx2B chimera (Figure 1D) [78]. This design aimed to preserve Stx2B in its native pentameric form, effectively mimicking the Gb3 binding sites of the full-length toxin. The StxB monomer was linked from its C-terminus to the N-terminus of the BLS monomer using a glycine/serine (G/S) flexible peptide linker, widely employed in protein engineering for its ability to maintain structural integrity, minimize steric hindrance, and preserve functional properties. We experimentally evaluated three different constructs containing linkers of five (GSGSG), ten (GSGSGSGSGS), and fifteen (GSGSGSGSGSGSGSG) amino acids in length. Based on extensive biophysical and immunological analyses [79], the construct bearing the G/S decapeptide linker was identified as the best candidate.

In contrast to the tendency of isolated StxBs to dissociate from their pentameric assemblies, in the chimeric structure, they remain associated. The stable oligomeric scaffold of BLS provides a supportive framework, effectively substituting for the role of the α-helix from StxA, which naturally inserts into the center of the pentamer in native toxins [74]. However, this BLS-assisted chimeric assembly is not rigid; our results demonstrate that the StxBs can move significantly as a whole due to the flexibility of the linkers connecting the two proteins. This highlights the dynamic behavior of the regions involved in eliciting neutralizing antibodies (Figure 1).

Thus, the BLS-Stx2B immunogen showed a remarkable capacity to elicit strong and long-lasting humoral immune responses against high lethal doses of Stx2 and its variants in mice, inducing highly neutralizing antibodies [78,80,81]. Most importantly, sera from immunized mice also protected mice in an STEC infection model, demonstrating that the transferred antibodies could neutralize the toxin as it is delivered by the bacteria [82]. Prompted by these results, we also designed a second immunogen combining the B subunit of Stx1 (Stx1B) with BLS, resulting in the BLS-Stx1B chimera, with the aim of developing an antibody therapy with broader neutralizing capacity against different variants of Stx.

### 4.3. BLS-StxB Chimeras Are Highly Immunogenic and Trigger Protective Antibody Responses Against STEC-HUS

**Figure 2:**
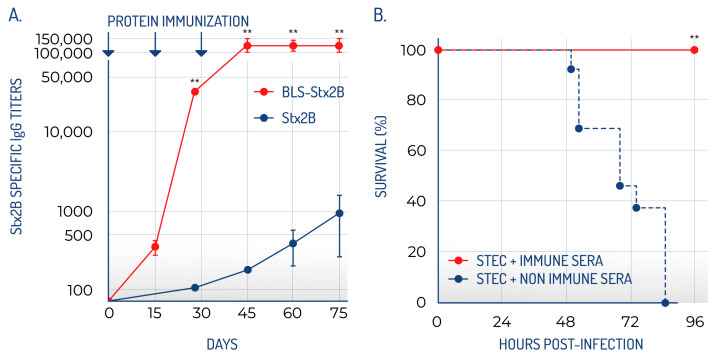
BLS-StxB chimeras are highly immunogenic and generate high titers of neutralizing antibodies. (**A**) The antibody titers generated by BLS-Stx2B are significantly higher than those of the Stx2B. (**B**) BLS-StxB immunized mice are protected against STEC infection (adapted from Mejías et al., 2013 [78]). ** statistically significant.

In a mouse model, BLS-Stx2B elicited significantly higher specific ELISA titers and enhanced neutralization activity compared to Stx2B alone (Figure 2A). While the Stx2B pentamer is only marginally stable in the absence of the A subunit, fusion to BLS strongly increases its stability, allowing the development of antibodies against the native conformation of the pentamer. Additionally, the antibodies produced by this chimera exhibited comparable neutralizing efficacy against both wild-type Stx2 and its variants. Previous studies have shown that all Gb3 binding sites within this toxin family are situated on the same side of the B pentamer, directly opposite the A subunit. Two of the three binding sites are formed by residues from adjacent monomers, requiring proper pentamer assembly [72]. Consequently, the ability of sera to neutralize various Stx family members is largely attributed to antibodies that recognize these conserved binding sites [84], which are present only when the B subunit assumes the correct pentameric structure [74]. This characteristic is crucial for the prevention and treatment of HUS, as antibodies that exhibit broad reactivity against Gb3 binding sites are likely to be highly effective in mitigating the damage caused by the Stx family.

Notably, immunization with BLS-Stx2B provided significant protection against the toxin in both in vitro and ex vivo studies, with all mice vaccinated with the BLS-Stx2B chimera successfully protected against Stx2 exposure (Figure 2B). Crucially, sera from these immunized mice also conferred protection in a relevant model of STEC infection, indicating that the transferred antibodies are capable of neutralizing the toxin as it is released by STEC. Mice that received the immune sera did not display any typical signs of STEC infection, such as symptoms of illness, renal impairment, increased neutrophil counts, or death. Additionally, the observation that immune sera diminished STEC colonization in the intestines implies that anti-Stx2B antibodies might effectively neutralize Stx2 locally. These findings led us to suggest that BLS-Stx2B could serve as a potential vaccine for HUS. Maternal immunization of mice with BLS-Stx2B proved highly effective in facilitating the transfer of specific antibodies, indicating that prior exposure of adult females to this immunogen could offer protection to their offspring during the early stages of life [80].

**Figure 3:**
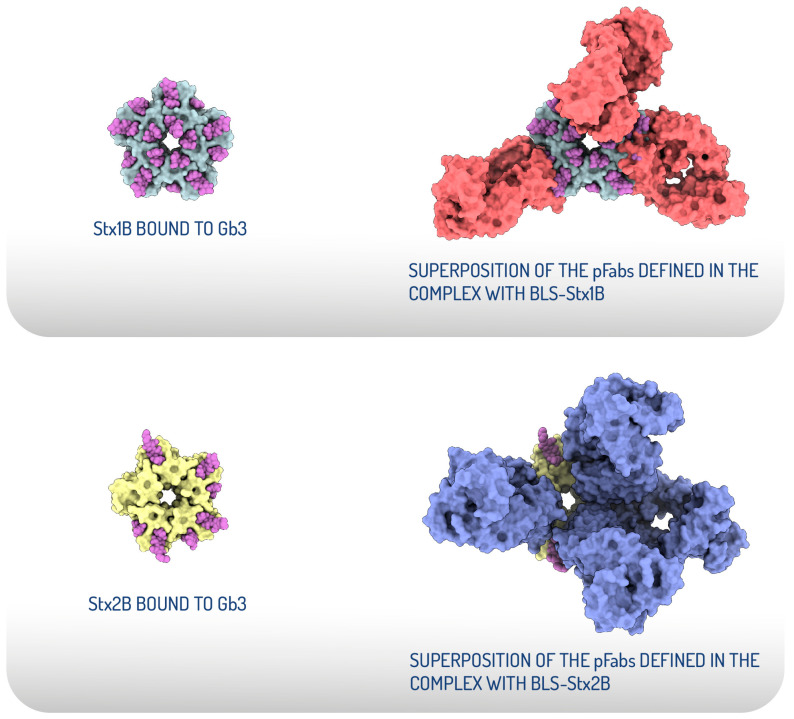
Structural comparison of the receptor-binding sites of Stx1B and Stx2B with the epitopes recognized by the pFabs derived from hyperimmunization with the chimeras. Top view of the crystallographic models displayed as surface representations of Stx1B in grey and Stx2B in yellow, both bound to Gb3 oligosaccharides, depicted as magenta spheres (**left**). Structural superposition of the crystallographic StxB/Gb3 complexes with the atomic coordinates of the pFab models. The pFab fragments are shown as surface representations in red (complex with BLS-Stx1B) and blue (complex with BLS-Stx2B) (**right**) (adapted from Cristofalo et al., 2025 [83]).

The cryo-EM structural analysis of BLS-Stx1B and BLS-Stx2B chimeras confirmed that these engineered immunogens effectively stabilize the StxB pentamers [83]. Both chimeric constructs exhibit high flexibility at their extremes, corresponding to motions of the StxBs with respect to the BLS core. Strikingly, structural evidence of the interaction between the chimeras and polyclonal Fab fragments demonstrates that the elicited neutralizing antibodies block most of the interaction surface of the toxins with their cellular receptors (Figure 3). These findings add structural evidence to validate BLS-Stx1B and BLS-Stx2B chimeras as potential candidates for developing a human vaccine and demonstrate the potential of an antibody-based therapy for mitigating STEC-HUS.

### 4.4. How Can We Take Advantage of the Extraordinary Immunogenicity of BLS-StxB Chimeras?

As stated previously, StxBs are the primary targets for immunotherapeutic interventions aimed at preventing or ameliorating the clinical manifestations of STEC-HUS. Vaccination with BLS-StxB chimeras would be the most effective approach to leverage their immunogenicity. Vaccinating pregnant women and children around one year of age may help prevent the development of HUS in STEC-infected children, as suggested by preclinical results [78,80,82]. However, developing a vaccine based on BLS-StxB chimeras would be a lengthy process [85], and a more rapid solution is needed for a condition that currently has no treatment. In contrast to vaccines, passive immunotherapies based on antibodies against Stxs are more feasible strategies against STEC-HUS. Specific therapies with monoclonal antibodies (mAbs) that target Stx and prevent its internalization through its specific receptor Gb3 were previously evaluated with promising preclinical results, but thus far, there is no evidence of conclusive effectiveness from clinical trials [86,87]. Single-chain antibodies (V_H_H) produced by camelids exhibit several advantages in comparison with conventional antibodies, making them promising tools for therapy. We exploited the immunogenicity of BLS-Stx2B to develop V_H_Hs with therapeutic potential against STEC-HUS. We identified a family of V_H_Hs against the Stx2B that neutralize Stx2 in vitro at sub-nanomolar concentrations. One V_H_H was selected and was engineered and presented with extended in vivo half-life and high therapeutic activity, as demonstrated in three different mouse models of Stx2-toxicity [81]. Even though this molecule appeared as a therapeutic option for treating STEC-HUS, only very few molecules of this class have been approved and reached the market in the last decade, and none against infectious diseases [88]. Thus, we decided to seek another strategy.

Polyclonal antibodies (pAbs), especially equine polyclonal antibodies (EpAbs), offer a promising therapeutic alternative [89]. Their safety and efficacy have been well established in cases of rabies virus and botulinum toxin exposure, as well as in treating bites from venomous snakes and scorpions [90,91,92,93]. Additionally, EpAbs have several advantages compared to mAbs. Firstly, they consist of F(ab’)_2_ fragments produced through pepsin digestion, which maintain the bivalent binding ability of IgG immunoglobulins while lacking the constant region (Fc) that may lead to Fc-mediated side effects. Secondly, EpAbs can recognize a broader range of epitopes and often exhibit higher avidity for their specific antigens than mAbs. They are also able to recognize various pathogen variants, venoms, or toxins, which reduces the likelihood of escape mutations and facilitates the rapid development and production of effective treatments.

With a focus on obtaining a therapy with broad neutralizing capacity against different STEC strains, we immunized horses with a mixture of BLS-Stx1B and BLS-Stx2B chimeras. These proteins were highly immunogenic in horses, eliciting very high titers of neutralizing antibodies against their cognate holotoxins. The sera showed a broad spectrum of neutralizing capacity against several variants of Stx in the preclinical models evaluated and, remarkably, protected against the STEC infection.

Building on these findings, we created INM004, an innovative therapy made up of F(ab’)_2_ fragments derived from equine immunoglobulins. This therapy efficiently neutralizes eight variants of Stx1 (Stx1a, Stx1c, Stx1d) and Stx2 (Stx2a, Stx2b, Stx2c, Stx2d, Stx2e) both in vitro and in vivo in an animal model of STEC-HUS, with the potential to prevent the development of HUS [82,94]. No significant toxicity was observed in preclinical studies, both in single-dose and repeated-dose toxicity studies in mice and rabbits [82]. INM004 demonstrated safety in animals that received higher doses or a greater number of injections than what is intended for human clinical trials or therapeutic use. The preclinical findings supported the initiation of a first-in-human clinical study of INM004 to evaluate its safety, tolerance, and pharmacokinetic characteristics.

## 5. Clinical Development of INM004: How to Deal with a Rare Disease

Orphan diseases are rare conditions affecting only a small proportion of the population. Orphan drugs are those designated by regulatory agencies such as the U.S. Food and Drug Administration (FDA) and the European Medicines Agency (EMA) as treatment approaches for orphan diseases. Developing drugs for rare diseases is more complex than for common diseases, as identifying suitable patients for clinical trials might be challenging [95,96,97]. Historical control data describing the natural history of rare diseases play a major role in developing treatments for orphan diseases where randomized, placebo-controlled trials may not be feasible (see Section 5.3) [98,99]. The FDA has the authority to grant orphan drug designation to a drug or biological product intended for the prevention, diagnosis or treatment of a rare disease [100,101,102]. Considering all these factors, we decided to seek scientific advice from different regulatory authorities to develop a clinical plan. As a result, we received advice from the Multidisciplinary Innovation Support Team of ANMAT (Argentinian Regulatory Agency), aimed at providing assistance to projects and products that are innovative and of interest to public health [103]. Moreover, INM004 was granted the Orphan Drug designation from the FDA and the EMA. INM004 also received the Rare Pediatric Disease (RPD) designation from the FDA, an additional incentive program specific to rare diseases in children [104,105].

### 5.1. Phase 1 Clinical Trial in Healthy Adult Volunteers

The objective of this phase 1 study was to assess the safety, tolerance, and pharmacokinetics (PK) of INM004 in human participants. Additionally, we examined the Stx-neutralizing ability of INM004 in the plasma of these volunteers. We carried out a single-center, randomized, phase 1, single-blind, placebo-controlled clinical trial to assess the safety, tolerance, and pharmacokinetics of INM004 in two stages. In stage I, participants received a single dose at two different levels (2 and 4 mg/kg), while in stage II, three consecutive daily doses of 4 mg/kg were administered. The study included healthy adult volunteers and took place from December 2017 to September 2018. Eligible participants included 14 healthy volunteers aged 18–55 years, with a body mass index (BMI) of 19–27 kg/m^2^, normal laboratory values, chest X-ray and electrocardiogram recordings. All participants provided written informed consent after receiving comprehensive information on the objectives, methods and potential harms related to their participation in the study. The study was approved by the local ethics committee and ANMAT. The study was prospectively registered in clinicaltrials.gov (NCT03388216). The infusion of a single and repeated dose was not associated with major side effects.

Infusions of INM004 did not lead to any serious or severe adverse events; only mild to moderate infusion-related adverse effects were noted, primarily including headache, flushing, and rhinitis. These effects were temporary and resolved without any lasting consequences. There were no reports of hypersensitivity reactions or issues at the injection site. INM004 infusions were well-tolerated in all stages of the study. Laboratory parameters presented transient minor variations without clinical significance, and there was no evidence of immunogenicity against the product in any of the healthy volunteers. The 2 mg/kg cohort effectively demonstrated in vitro toxin neutralization, even when considering this as the most difficult therapeutic situation. As a result, we moved forward with exploring a dosage of 4 mg/kg, expecting an enhanced neutralizing ability. The resultant data proved sufficiently robust to warrant the initiation of a phase 2 clinical trial involving pediatric patients and using a 4 mg/kg dose, factoring in the scenario with the highest antibody concentration [106].

### 5.2. Phase 2 of INM004 for Preventing the Development of HUS in STEC-Infected Patients: An Ideal but Difficult Goal

The development of a drug to treat a rare disease like STEC-HUS presents a significant challenge. The two main difficulties are the lack of knowledge about the therapeutic window in which Stx-neutralizing antibodies would be more effective and the low incidence of STEC infections vs. the number of patients required to demonstrate efficacy. It is tempting to think that the sooner the treatment is applied, the better the clinical evolution of STEC-HUS-affected patients would be. The goal of administering INM004 is to prevent the onset of HUS by inhibiting the circulating toxins in individuals infected with STEC. Thus, INM004 may be applicable for patients presenting with bloody diarrhea and a positive Stx2 test result, as they are at an increased risk of developing HUS [5]. A prompt interruption of the Stx-mediated pathway is anticipated to hinder the progression of HUS, reduce the severity of the disease, lower the rate of complications, and shorten both the incidence and duration of hospital stays. Consequently, patients in the early stages of the disease were the target of our first Phase 2/3 clinical trial, i.e., children who sought medical care due to diarrhea associated with STEC infection before HUS development.

We designed a double-blind, placebo-controlled, adaptive, phase 2/3 study to evaluate the pharmacokinetics, safety, and efficacy of INM004 in pediatric patients with Stx2-positive bloody diarrhea for the prevention of HUS. Pediatric subjects between 1 and 10 years of age at the time of screening, with an increased risk for the development of HUS, were enrolled. Bloody diarrhea and a positive screen for Stx2 were used as inclusion criteria, as these factors have been identified as risk factors for STEC-HUS development and were thought to serve to enrich the patient population within the study with those most likely to benefit from this therapy. The primary objective of this study was to demonstrate the efficacy of INM004 as measured by the prevention of HUS development at 4 weeks after the first dose of the study drug compared to placebo in addition to Standard of Care (SoC). A clinical trial was conducted in 16 centers across Argentina, and only 11 patients were randomized during the active enrollment period (October 2019 to March 2020). Of the eleven randomized patients, four were male and seven were female. Four patients were assigned to the “Placebo” treatment arm, four to the “low level INM004” (one dose), and three to “high level INM004” (two doses) treatment arms. In March 2020, enrollment was suspended due to the pandemic caused by the SARS-CoV-2 virus. In May 2022, we decided to end the clinical trial. During that period, we analyzed the reasons for the very low recruitment in this trial. The prevention strategy of STEC-HUS using INM004, although it would be presented as optimal from the pathophysiology of the disease, was unfeasible. Most patients with bloody diarrhea attend health centers of low complexity or remain at home and are managed as outpatients. When symptoms worsen, patients go to tertiary care centers (either by spontaneous demand or referral), where HUS diagnosis is established, and the patients are hospitalized. Therefore, it is already too late for the implementation of a prevention strategy. Given these difficulties in the recruitment and the rate of progression of pediatric patients with Stx-positive bloody diarrhea to STEC-HUS (10–15%), it would take more than ten years to recruit 400 patients to demonstrate the efficacy of INM004 in preventing the onset of HUS. These results show that a preventive trial is clearly an impossible goal in real-world conditions.

### 5.3. Phase 2 Assessing the Safety, Pharmacokinetics and Efficacy of INM004 in Pediatric Patients with STEC-HUS

As mentioned above, a child with diarrhea or bloody diarrhea, vomiting, abdominal pain, and decreased urine output, is usually attended to at a tertiary care center (either by spontaneous demand or referral), where the HUS diagnosis is established, and the patient is hospitalized. Since it has been demonstrated that Shiga toxin is present in circulation at the early stages of STEC-HUS [49], there is still an opportunity for treatment with neutralizing antibodies. Thus, we proposed a phase 2 trial to test the intervention with INM004 in hospitalized patients with a diagnosis compatible with STEC-HUS. The objective of this study was to evaluate the efficacy, safety, and pharmacokinetics of INM004 administered to subjects who were within 13 days of the diarrhea onset at the time of diagnosis of a condition compatible with STEC-HUS. We chose this timeframe because, although the median time to development of HUS is 7 days from the diarrhea onset, there are patients with more abrupt evolution, approximately 5 days, and others with a later presentation, up to 13 days [41].

**Figure 4:**
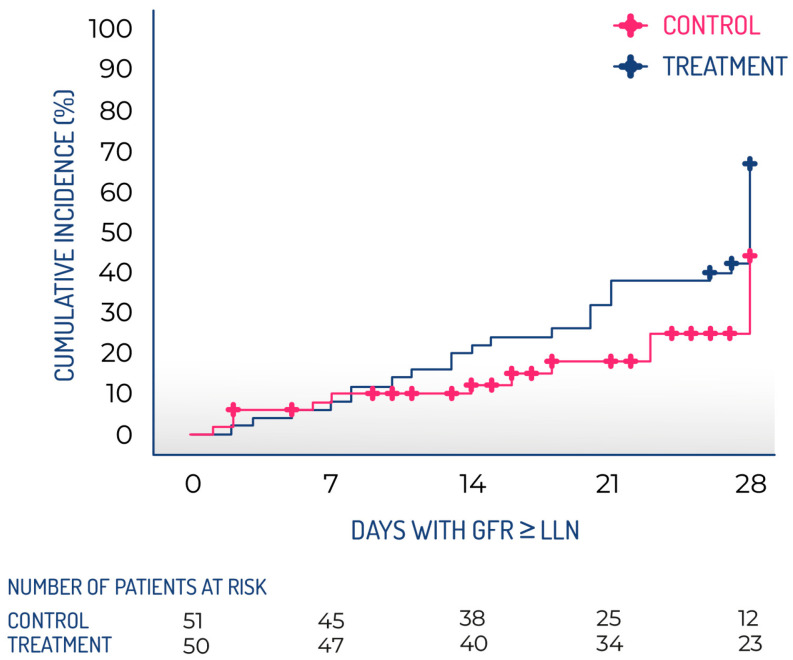
Kaplan–Meier cumulative incidence of glomerular filtration rate recovery during the follow-up period. GFR, glomerular filtration rate; LLN, lower limit of normal.

This study aimed to shed light on the safety and therapeutic potential of INM004, paving the way for a future phase 3 trial. A multicenter, open-label phase 2 clinical trial of INM004 was conducted with a historical control group in 16 reference hospitals throughout Argentina. Participants eligible for the treatment group were patients diagnosed with STEC-HUS who were hospitalized at the participating centers between September 2022 and May 2023 (during the warm season). The control group consisted of patients from the same seasonal periods in 2018–2019 and 2019–2020, prior to the COVID pandemic, identified through a systematic review of clinical records at each participating center. The choice of an open clinical trial design controlled with a historical group [107,108] was made following the synthetic control methods accepted by both the FDA and the European Medicines Agency [104,109]. This study design allowed for the enrollment of more patients in the treatment arm in the short term. The same selection criteria were applied to both arms, and the only difference was the treatment applied (INM004 plus SoC for STEC-HUS in the treatment group vs. only SoC in the historical group). Given that the standard of treatment for these patients has not changed in recent years, and that the controls were taken from the same participating centers, it was expected that the two groups would be comparable in terms of other variables of interest. The non-randomized design may have introduced a potential source of bias. To reduce selection bias, several strategies were implemented in both the study design (including the same centers, enrollment during the same seasonal period, consistent eligibility criteria, and identical SoC for both the treatment and control groups; as well as a systematic review of clinical records for selecting subjects in the control group) and the statistical analysis (utilizing a propensity score matching procedure). To mitigate the possible bias from the unblinded design, objective variables were chosen as efficacy endpoints. The primary objectives of this study were to evaluate the efficacy of INM004, in addition to SoC, in improving renal involvement in patients with STEC-HUS; to assess the safety of INM004 administration in the treatment group; and to assess the PK of INM004 in the treatment group. This study was considered to be the best way to determine the efficacy endpoints for the future confirmatory phase 3 study. A total of 57 patients were included in the treatment group, while 125 patients were part of the control group. Following the propensity score matching process, 52 patients remained in each group. The research demonstrated that INM004 can be safely given to pediatric patients suffering from STEC-HUS and provided PK data that validated the proposed regimen of two doses at 4 mg/kg for this demographic group. Additionally, the study indicated non-statistically significant trends suggesting improvement in kidney function during the acute phase of STEC-HUS. In the primary efficacy endpoint, patients in the treatment arm presented a non-statistically significant difference of two dialysis days. On secondary endpoints, non-statistically significant trends toward fewer patients needing dialysis and fewer patients requiring dialysis for more than 10 days, as well as a shorter time to GFR normalization, were observed, favoring the treatment arm (Figure 4). These results described by Fayad et al. [110] allowed us to move forward to a phase 3 study to evaluate the efficacy of INM004 in the treatment of STEC-HUS.

### 5.4. A Phase 3 Study to Evaluate the Efficacy of INM004 in Pediatric Patients with STEC-HUS

Having completed phases 1 and 2 of the study of INM004 and given the need to confirm efficacy and to complete PK data, a phase 3 clinical study was proposed. A multicenter, competitive, randomized, double-blind, adaptive, placebo-controlled clinical trial design was chosen, and the study is currently underway. The study population includes subjects between 9 months and 18 years old who were hospitalized for STEC-HUS. Although the highest incidence of STEC-HUS is in populations between 1 and 5 years old, cases reported in children under one year and adolescents are not negligible, both in Argentina and in other regions of the world.

**Figure 5:**
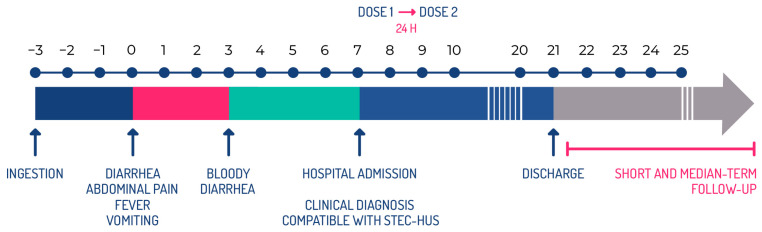
Therapeutic intervention scheme according to the evolution of the disease during the acute phase.

In this phase 3 study, it is proposed to perform the intervention with INM004 in patients diagnosed with STEC-HUS who require hospitalization for their care and treatment. Figure 5 shows a therapeutic intervention scheme considering the mean time of evolution from the STEC-HUS infection during the acute phase.

Treatment with INM004 should be started as soon as possible after the diagnosis of the disease. For this reason, in this clinical trial, the first dose of INM004 is being administered within 6 h of the signing of the informed consent, in patients with a maximum of 10 days from the onset of diarrhea and no more than 24 h from the time of diagnosis of STEC-HUS in the participating center. The rationale for choosing the 10-day time frame from the onset of diarrhea is that, although HUS generally manifests between days 5 and 14 of the onset of diarrhea, evidence of microangiopathic involvement is present by days 8–9, and anuria, if it occurs, rarely begins after day 10 [5]. This time limit is then proposed, where it is expected that, according to the pathophysiology of the disease, there will still be toxin circulation.

**Figure 6:**
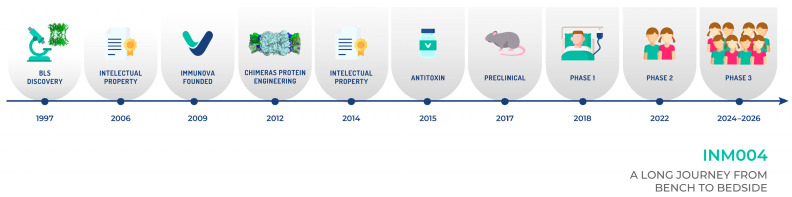
A timeline of the process to develop INM004 is depicted. The basic research stage took more than 15 years, while the clinical stage will take about ten years. Overall, this timeline shows how difficult it is and how much effort is needed to develop a potential drug for a neglected disease.

The main objectives of this study include evaluating the efficacy of INM004 in improving renal function during the acute phase of STEC-HUS. Although the degree of renal damage associated with STEC-HUS is variable, it has been reported that up to 60% of affected patients show severe forms of AKI with prolonged periods of oligoanuria and dialysis requirements, and that about 30% of patients develop chronic renal sequelae [4,111]. In recent decades, the concept of AKI, defined as an increase in serum creatinine or a decrease in GFR, has gained relevance in predicting the evolution to chronic kidney disease, both in medical practice and in clinical studies [112,113,114,115]. In the phase 2 study, where patients were followed for 28 days after STEC-HUS diagnosis, a trend towards a shorter time to recovery of renal function (defined as the time until reaching a GFR ≥ LLN (lower limit normal) for age and sex in the absence of dialysis) was observed in the group treated with INM004. Thus, the time to recovery of renal function during the early phase of STEC-HUS was proposed as an appropriate endpoint to evaluate the efficacy of INM004. The objective of the intervention with INM004 is the amelioration of renal function during the acute phase of the disease, considering that a shortening of renal damage will imply fewer days of AKI, and prevention or reduction of the days of dialysis and anuria and therefore a better prognosis. Being a continuous variable, time to recovery allows for detecting differences between groups with a smaller number of subjects than necessary if dichotomous variables are used. In this way, the estimated sample size (two parallel arms with a total of 220 patients randomized 1:1 to two doses of placebo and INM004) is reachable in a period of three years, which is of great importance in the context of this orphan disease. This phase 3 trial was launched in October 2024, and 22 participating sites were included in Argentina, which are specialized in the management of pediatric STEC-HUS. Additionally, 20 centers from eight European countries (France, the United Kingdom, Spain, Italy, Germany, Ireland, Belgium and the Netherlands) are also participating. By the time of submitting this article, more than 20% (47/220) of the recruitment had been achieved, and the expectation is to finish the trial in the next two years (Figure 6). In summary, the ongoing phase 3 trial has been planned to demonstrate the efficacy of INM004 as a specific therapy for the treatment of hospitalized pediatric patients with STEC-HUS during the acute phase. INM004 is expected to ameliorate kidney damage by disrupting the Stx-mediated cascade, with a faster recovery of renal function, which would improve morbidity and mortality.

## 6. Potential Extensions of Indication for INM004

Should we be able to demonstrate INM004’s efficacy in a therapeutic setting, we believe that the efficacy of the preventive indication can be revisited using a different strategy. Clearly, the existence of a specific drug to treat STEC-HUS can serve to modify the SoC and allow for the early hospitalization of patients with a clinical manifestation of bloody diarrhea and a Stx-positive result.

In the same sense, the indications of INM004 could be extended to adults. The phase 3 protocol includes patients up to 18 years for several reasons related to the feasibility of the trial and the number of patients needed to demonstrate efficacy. STEC-HUS is rarer among adults than in children but causes more severe disease and death. The underlying conditions, especially immunodeficiency, are strongly associated with decreased survival. For these reasons, the current phase 3 study cannot include adults, as baseline medical conditions, disease evolution, and SoC might not be comparable between children and adults. Thus, after finishing the current trial, different strategies can be discussed with the regulatory agencies to develop a clinical plan to demonstrate the efficacy of INM004 as a preventive and/or therapeutic drug for STEC-HUS in adults.

## 7. Development of a Vaccine for Prevention of STEC-HUS

A vaccine for STEC-HUS should be considered an orphan vaccine, with the difficulties and barriers already explained. However, a clinical demonstration of efficacy of INM004 to ameliorate the effects of this syndrome would constitute a very strong proof of principle and an immune correlate of protection for a vaccine based on BLS-StxBs chimeras. Vaccinated pregnant women and children should be appropriately protected against STEC-HUS by this vaccine. Should we succeed in our phase 3 trial, we will attempt to develop a preventive vaccine for this disease.

## 8. Etiological Diagnosis

HUS is diagnosed clinically with laboratory tests that establish non-immune hemolytic anemia, thrombocytopenia, and abnormal renal function. However, the etiological diagnosis is important to differentiate STEC-HUS from complement-mediated HUS, due to the differences in treatment and outcome [116]. STEC isolation is the gold standard for diagnosis. Stool samples should be obtained as soon as possible after diarrhea onset, because the bacterial load decreases shortly after disease onset. Rectal swab samples should only be used as an alternative if the patient does not present with catharsis. On the other hand, detection of serum antibodies to the LPS of STEC is a valuable diagnostic tool, given that these antibodies persist for several weeks [117,118]. Historically, in Argentina, the evidence of STEC infection in HUS patients was around 30%. However, the incorporation of an indirect ELISA (Glyco-iELISA) for the detection of IgM and IgG antibodies against the O polysaccharide of the LPS of *E. coli* O157, O145 and O121 serogroups in 2016 improved the diagnostic performance, reaching >70% [119,120,121].

Moreover, the recent development of an *E. coli* O157/O145 LFIA (lateral flow immunoassay) has allowed the detection of specific IgM antibodies very early in the course of the infection, making it an ideal diagnostic tool to be implemented in pediatric emergencies and, thus, avoid delays in the application of the correct supportive or specific treatment and prevent complications associated with HUS [122]. In the phase 2 trial of INM004, STEC infection was diagnosed following SoC procedures at each site for both arms (including detection of Stx by enzyme immunoassay (EIA) or detection of *stx* genes by PCR in stool, fecal culture for *E. coli* O157, and/or detection of specific anti-O polysaccharide IgM/IgG antibodies against *E. coli* O157, O145, O121 and O103 serogroups using the commercial kit CHEMLIS^®^ E. coli Combi Glyco-iELISA -Chemtest Argentina S. A.-). For the treatment arm, the serological analysis was repeated using the CHEMLIS^®^ E. coli Combi Glyco-iELISA kit and was completed by analyzing the specific anti-O polysaccharide IgM/IgG antibody response against the *E. coli* O111, O26 and O45 serogroups in 55/57 patients with serum samples [123].

STEC diagnosis was confirmed in 55 (96.5%) patients in the treatment group and 102 (81.6%) in the control group, with O157 being the most frequent serogroup (59.6% and 44.0%, respectively), followed by O145 (26.3% and 15.2%). Stx or its genes were identified in 37 (64.9%) patients in the treatment group and 78 (62.4%) in the control group. Among these patients, genotype was determined in over 70%: *stx*_2_ was found in all patients, either alone (88.9% in treated vs. 94.6% in controls) or in combination with *stx*_1_ (11.1% vs. 5.4%). The STEC serogroup was identified in 52/57 (91.2%) patients in the treatment group, whereas in the control group, the proportion was lower (64.8%). In 31.6% of patients in the treatment group and 16.8% in the control group, the diagnosis was confirmed only by the detection of anti-polysaccharide antibodies.

The STEC-HUS cases studied during the clinical trial presented the same characteristics observed in Argentina in recent years, in relation to the clinical presentation, the serogroups detected, and the predominant *stx* genotype. STEC diagnosis improved with the incorporation of antibody detection, reaching 96.5% of the patients studied. Serogroup O157 continues to be prevalent in Argentina as well as in other regions, while O145 has been identified as the second most frequent serogroup in the country [124]. This serogroup has also been reported in patients with STEC-HUS from several countries in Europe [11] and the U.S. [125], although non-O157 infections may be underestimated because of diagnostic limitations [117]. Currently, for the phase 3 study under development, serum samples from all the patients are being collected for the detection of IgM antibodies against *E. coli* O157, O145, O121, and O103 by CHEMLIS^®^ E. coli Combi Glyco-iELISA. Furthermore, the *E. coli* O157/O145 LFIA (CHEMSTRIP^®^ *E. coli* O157/O145), ref. [122] has been implemented at the centers in Argentina, as an early screening test for the rapid diagnosis of STEC infection. In addition, stool samples are being analyzed by RT-PCR for the detection of *stx*, *eae,* and serogroup-specific genes (O157 and the big six: O26, O45, O103, O111, O121 and O145). If INM004 treatment demonstrates efficacy, these newly developed diagnostic tools would be crucial to accelerating the extension of the indication from therapeutic to preventive use of this immunotherapy. A more efficient early diagnosis could be very important for the treatment of patients and contacts during outbreaks.

## 9. Conclusions

INM004 immunotherapy could represent a new step in the treatment of STEC-HUS after admission, hopefully preventing long-term damage [126]. Earlier and broader use of INM004 is envisaged if phase 3 turns out to be successful, helping to diminish the burden of STEC-HUS. The use of INM004 therapy, focused vaccination strategies, and new diagnostic tools can change the impact of this endemic disease.

## Figures and Tables

**Table 1:** HUS/100,000 children < 5 years.

Country	HUS	Reference
Argentina	6.03	[17]
Belgium	3.18	[18]
Czechia	2.33	[18]
Italy	1.57	[19]
The United Kingdom and Ireland	1.54	[20]
Sweden	1.53	[18]
United States	1.22	[21]
Canada	1.04	[22]
Germany	1.03	[18]
Romania	0.72	[18]
Poland	0.70	[18]
Australia	0.49	[12]
Portugal	0.49	[18]
Norway	0.36	[18]
Netherlands	0.23	[18]

## Data Availability

No new data were created or analyzed in this study.
